# DNA defects, epigenetics, and gene expression in cancer-adjacent breast: a study from The Cancer Genome Atlas

**DOI:** 10.1038/npjbcancer.2016.7

**Published:** 2016-05-04

**Authors:** Melissa A Troester, Katherine A Hoadley, Monica D’Arcy, Andrew D Cherniack, Chip Stewart, Daniel C Koboldt, A Gordon Robertson, Swapna Mahurkar, Hui Shen, Matthew D Wilkerson, Rupninder Sandhu, Nicole B Johnson, Kimberly H Allison, Andrew H Beck, Christina Yau, Jay Bowen, Margi Sheth, E Shelley Hwang, Charles M Perou, Peter W Laird, Li Ding, Christopher C Benz

**Affiliations:** 1 Lineberger Comprehensive Cancer Center, University of North Carolina at Chapel Hill, Chapel Hill, NC, USA; 2 Department of Epidemiology, University of North Carolina at Chapel Hill, Chapel Hill, NC, USA; 3 Department of Pathology and Laboratory Medicine, University of North Carolina at Chapel Hill, Chapel Hill, NC, USA; 4 Department of Genetics, University of North Carolina at Chapel Hill, Chapel Hill, NC, USA; 5 The Eli and Edythe L. Broad Institute of MIT and Harvard, Cambridge, MA, USA; 6 The McDonnell Genome Institute, Washington University, St Louis, MO, USA; 7 Canada’s Michael Smith Genome Sciences Centre, BC Cancer Agency, Vancouver, BC, Canada; 8 USC Epigenome Center, University of Southern California, Los Angeles, CA, USA; 9 Center for Epigenetics, Van Andel Research Institute, Grand Rapids, MI, USA; 10 Department of Pathology, Division of Anatomical Pathology, Beth Isreal Deaconess Medical Center and Harvard Medical School, Boston, MA, USA; 11 Department of Pathology, Stanford University School of Medicine, Stanford, CA, USA; 12 Department of Pathology, Harvard Medical School, Boston, MA, USA; 13 Buck Institute for Research on Aging, Novato, CA, USA; 14 The Research Institute at Nationwide Children’s Hospital, Columbus, OH, USA; 15 National Cancer Institute, Rockville, MD, USA; 16 Department of Surgery, Duke University Comprehensive Cancer Center, Durham, NC, USA; 17 Department of Genetics, Washington University, St Louis, MO, USA

## Abstract

Recurrence rates after breast-conserving therapy may depend on genomic characteristics of cancer-adjacent, benign-appearing tissue. Studies have not evaluated recurrence in association with multiple genomic characteristics of cancer-adjacent breast tissue. To estimate the prevalence of DNA defects and RNA expression subtypes in cancer-adjacent, benign-appearing breast tissue at least 2 cm from the tumor margin, cancer-adjacent, pathologically well-characterized, benign-appearing breast tissue specimens from The Cancer Genome Atlas project were analyzed for DNA sequence, copy-number variation, DNA methylation, messenger RNA (mRNA) sequence, and mRNA/microRNA expression. Additional samples were also analyzed by at least one of these genomic data types and associations between genomic characteristics of normal tissue and overall survival were assessed. Approximately 40% of cancer-adjacent, benign-appearing tissues harbored genomic defects in DNA copy number, sequence, methylation, or in RNA sequence, although these defects did not significantly predict 10-year overall survival. Two mRNA/microRNA expression phenotypes were observed, including an active mRNA subtype that was identified in 40% of samples. Controlling for tumor characteristics and the presence of genomic defects, this active subtype was associated with significantly worse 10-year survival among estrogen receptor (ER)-positive cases. This multi-platform analysis of breast cancer-adjacent samples produced genomic findings consistent with current surgical margin guidelines, and provides evidence that extratumoral RNA expression patterns in cancer-adjacent tissue predict overall survival among patients with ER-positive disease.

## Introduction

Local recurrence risk has been hypothesized to arise from breast tumor multifocality and from genomic alterations in the benign-appearing cancer-adjacent tissue. Breast tumor multifocality may be as high as 40–60%,^1,2^ even for early stage (⩽T_2_) disease, where 2-cm surgical margins can leave tumor foci behind in up to 42% of cases.^3^ Genomic alterations in cancer-adjacent benign-appearing tissue are also prevalent.^4^ A previous study demonstrated somatic loss of heterozygosity in 6 out of 10 morphologically normal lobules adjacent to breast cancers.^5^ Subsequent studies showed shortened telomeric DNA in >50% of cases and four to five times more prevalent loss of heterozygosity within 1 cm of microscopically defined tumor margins.^6^ Defects can be far ranging, with methylation differences as far as 4 cm from such margins.^7^ Thus, genomic alterations in cancer-adjacent benign breast tissue may explain local recurrence rates, which range from 6 to 20% when breast-conserving therapy is accompanied by adjuvant therapy,^8,9^ but can exceed 40% when radiotherapy is not used with breast-conserving therapy.^10,11^ However, these peritumor field defects have never been significantly associated with patient survival and recent clinical trials and surgical guidelines suggest no benefit from wider surgical margins.^12^ The high prevalence of genetic and epigenetic field defects may be mitigated by radiotherapy.

In the decades since field cancerization was first reported, the availability of high-throughput methods for DNA and RNA analyses have changed dramatically, allowing cancer-adjacent tissue to be characterized more comprehensively, and avoiding the biases inherent in utilizing only a single genomics data type. On the basis of these methods, it is possible to identify the prevalence of any type of genomic defect, without the biases inherent in only utilizing a single platform. We performed RNA and DNA analyses on breast cancer-adjacent, benign-appearing tissue, sampled at least 2 cm from tumor margin. We then compared sequence data for tumors and adjacent normal tissues with blood to determine the somatic copy number and sequence changes. Evaluating multiple data types on such matched sets of samples allows a comprehensive picture of the genetic and epigenetic features of cancer-adjacent, benign-appearing tissue.

Beyond field effects, defined as defects in the genome of histologically normal epithelium, recent work has suggested that stromal characteristics of cancer-adjacent tissue may also affect progression, particularly among estrogen receptor (ER)-positive cases. Roman-Perez *et al.* ^13^ showed that there were two main expression subtypes in cancer-adjacent tissue and that one subtype, termed active, was associated with mortality. Subsequent research showed that this active subtype is commonly characterized by adipose-rich tissue,^14,15^ and several laboratories have shown that adipose tissue in breast may be infiltrated by CD68-positive immune/macrophage cells that are often arranged in crown-like structures.^16,17^ Most recently, the presence of CD68-positive cell complexes within ER-positive breast tumors was associated with the higher risk of developing distant metastatic disease, providing a link between these various studies.^18^ However, no study has examined peritumor microenvironment effects on patient survival while also controlling for the presence of DNA defects in the cancer-adjacent tissue.

Using multi-platform analysis of mutation, copy number, methylation, histology, and expression data, we estimated the prevalence of genomic defects and the expression phenotype of cancer-adjacent normal tissues, and then evaluated these findings in relation to overall patient survival.

## Results

### Evidence of genomic and pathological defects in cancer-adjacent tissue

**Figure 1:**
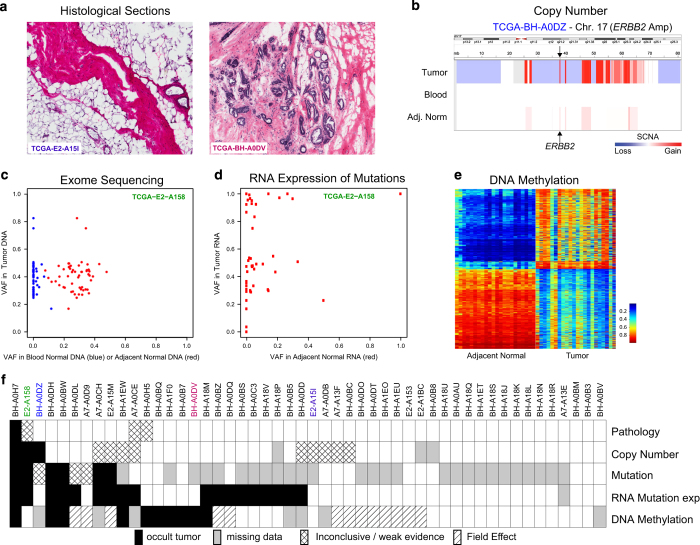
Multiple genomic assays demonstrate abnormalities within histologically normal, breast cancer-adjacent tissue samples. (**a**) Images of frozen sections of cancer-adjacent normal tissues (TCGA-E2-A25I and TCGA-BH-A0DV). (**b**) Example of DNA copy-number alterations visible in cancer-adjacent tissue (TCGA-BH-A0DZ). (**c**) Representative exome-sequencing comparing variant allele fraction of blood normal and cancer-adjacent normal versus tumor (TCGA-E2-A158). (**d**) Representative RNA sequencing comparing variant allele fraction cancer-adjacent normal versus tumor (TCGA-E2-A158). (**e**) Comparison of DNA methylation profiles between cancer-adjacent tissues and tumor tissues. (**f**) Genomic alterations in samples across multiple data platforms. Adj., adjacent; TCGA, The Cancer Genome Atlas Project; VAF, variant allele fraction.

All samples used to estimate prevalence of genomic alterations were evaluated by a pathologist at the Biospecimen Core Resource and a subset of 50 were re-evaluated by three pathologists who were blinded to both genomic and clinical data. Figure 1a shows the examples of images from frozen sections that were used to assess the presence of tumor cells and to confirm normal histology. Fifty samples of cancer-adjacent tissue had matching histology images, and among these, histological re-evaluation identified only a single sample with tumor cell foci among otherwise normal breast tissue components. Frozen tissue sections from three other samples were of insufficient quality to conclusively evaluate the presence or absence of microscopically detectable tumor cell foci.

Different DNA platforms showed variable sensitivity in identifying somatic abnormalities in the cancer-adjacent tissue samples. First, clear detectable copy-number alterations in these samples were rare, with 10% prevalence in the triplet samples (Table 1; Supplementary Table 3). An example of somatic DNA copy-number alterations in tumor and adjacent normal compared with blood from a single patient is shown in Figure 1b. Second, sequence defects in cancer-adjacent tissue DNA were much more common than copy-number alterations. Figure 1c shows exome-sequence analysis, in which 25% of cancer-adjacent samples had moderate-to-high levels of tumor-like somatic mutations (Table 1), although the variant allele fraction was low (typically <5%), consistent with low tumor cellularity (Supplementary Table 4). On average, about half of a tumor’s somatically mutated loci were expressed in matched cancer-adjacent normal tissue (sample mean 55%, minimum 10× read depth). Only 7% of RNA-expressed loci possessed the mutant allele detected by DNA sequencing (Figure 1d), but 44% of specimens had at least two detectable mutations by RNA-Seq (Table 1; Figure 1f, two or more variant reads; Supplementary Table 4B). Finally, for DNA methylation profiles (Figure 1e), we interpreted linear methylation patterns in the matched adjacent normal tissue as evidence of occult tumor cells (see Materials and Methods). No statistically significant correlation was found between median hypermethylation of 500 tumor-associated probes and tumor cellularity (Pearson correlation *P*-value=0.31, *r*=−0.09), suggesting that this approach was not confounded by cellularity. We identified tumor-like characteristics in ~15% of cancer-adjacent samples, whereas another 36% of samples showed methylation abnormalities suggestive of field cancerization (Supplementary Table 1; Supplementary Table 5). Although extraneous sources of variation between tumor and normal tissues (such as tissue composition differences) and temporal changes in the tumor’s epigenetic profile may confound such analyses, these methylation data suggest that up to 51% of cancer-adjacent tissues possessed either occult tumor cells or field cancerization effects.

We queried the normal tissue from 40 samples with paired tumor, looking for significantly mutated genes from our first The Cancer Genome Atlas project (TCGA) breast cancer manuscript.^19^ Thirty-one of the 40 paired tumors had mutations (*n*=53) representing 17 significantly mutated genes, including 18 PIK3CA mutations and 11 TP53 mutations. Among 18 PIK3CA mutations found in tumor, 11 were identified in the adjacent normal, but the maximum VAF was 3.52% and 6 were under 1%. This range of VAF values was much lower than that in tumors (from 13–60% with a median of 37% VAF). Similarly, among the 11 TP53 mutations found in tumor, 5 were present in adjacent normal, but only 1 had VAF >1% (compared with tumor VAF range 24–84%, median 44%).

Only one cancer-adjacent sample (BH-A0H7) showed genomic alterations across all DNA and RNA sequence assay platforms, and histological re-evaluation confirmed tumor cells infiltrating the adjacent breast tissue in this sample. RNA sequence and DNA results were not always consistent for a given sample. Among 49 samples that had no microscopic evidence of tumor cell foci, 15 (30%) or 21 (43%) showed strong evidence of genetic or epigenetic abnormality by at least one or two assay platforms, respectively (Figure 1f).

### Expression subtypes of cancer-adjacent, benign-appearing breast tissue

**Figure 2:**
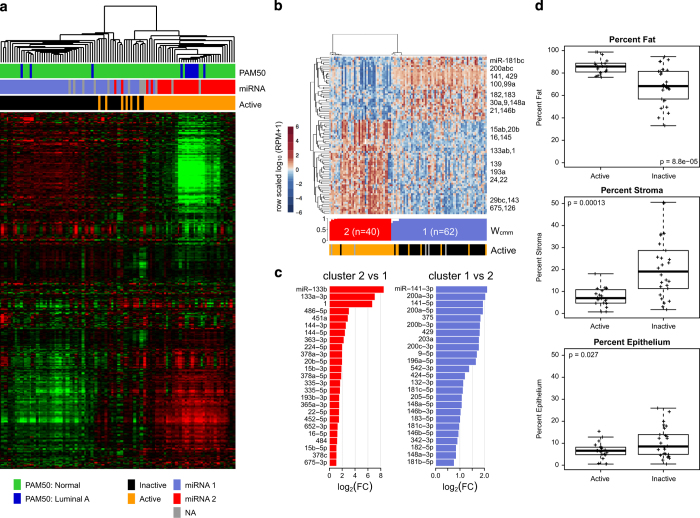
Expression characteristics of cancer-adjacent tissue samples. (**a**) Unsupervised gene expression clustering of RNA-sequencing data reveals two main clusters. Colored bars represent PAM50 subtype and miRNA subtype based on consensus cluster, and active/inactive subtype from Roman-Perez *et al.*^13^ (**b**) Normalized abundance heatmap for the a two-cluster NMF consensus clustering solution for microRNA profiles for 102 cancer-adjacent normals, for the 90 mature strands that had an absolute value fold change of at least 1.5 and a mean RPM of at least 25 in at least one of the two clusters. Below the heatmap, the silhouette width profile (*W*_cmm_) calculated from the consensus membership matrix. The covariate track shows samples scored as active versus inactive from mRNA data. (**c**) miRs that were differentially abundant (FDR<0.05) between two unsupervised clusters, for the largest 25 fold changes (FC), for miRs with FC>1.5 and a mean (RPM)>25 in at least one of the two clusters. (**d**) Association of mRNA clusters with fat, stromal, and epithelial percentages in normal tissues. FDR, false discovery rate; NMF, non-negative matrix factorization.

The dominant gene/microRNA expression variability among cancer-adjacent breast tissue samples reflected inter-individual differences in the breast microenvironment (unsupervised clustering shown in Figure 2; Supplementary Table 6). We evaluated tumor-like messenger RNA (mRNA) expression by applying a PAM50 tumor classifier.^20^ Although the majority of samples showed normal-like gene expression (Figure 2a), 12 out of 107 showed transcript profiles similar to luminal A breast cancers (Table 1). When we used miR data to cluster cancer-adjacent with matched tumor samples, 5 out 102 samples showed tumor-like miR profiles (Table 1) and clustered with tumors rather than cancer-adjacent samples (data not shown). However, tumor-like gene expression was not the dominant source of transcript variation among cancer-adjacent samples. Rather, unsupervised consensus clustering of the most variably expressed genes (3,280 mRNAs) returned two stable mRNA clusters (Figure 2a), which corresponded to the previously defined active and inactive gene expression subtypes found in histologically normal tissue adjacent to breast cancers.^13^ A two-cluster unsupervised consensus clustering solution for the most variably expressed microRNA mature strands (303 miRs) was highly (90%) concordant with the mRNA clusters (Figure 2b; Supplementary Figure 1). Mature strands that were differentially abundant between the two miR-based clusters included many that have been associated with breast cancer, including miR-1, miR-9, miR-133a, miR-196a, and miR-200 family members (q-value<0.001; Figure 2c; Supplementary Table 7). MicroRNA cluster 1 corresponded to inactive mRNA profiles, and had high stromal and epithelial content, with lower adiposity (as measured by histological area; Figure 2d).

### Association between genomic defects, expression subtypes, and survival

**Figure 3:**
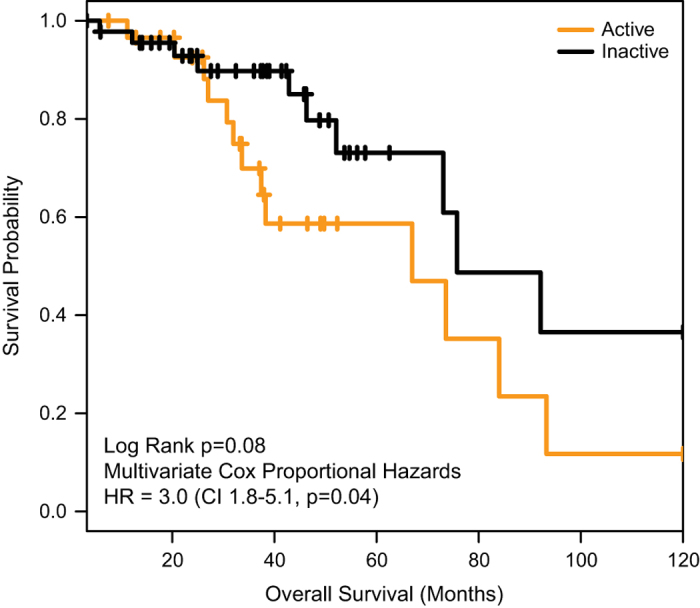
Overall survival is associated with active/inactive subtype among ER-positive patients. Kaplan–Meier curves show that patients with active microenvironment (*n*=46) have poorer survivorship than those with inactive microenvironment (*n*=30). Cox proportional hazards regression was used to estimate hazard ratios, adjusting for nodal status, intrinsic tumor subtype, tumor stage, tumor size, patient age in decade, and presence/absence of other cancer-adjacent genomic defects. ER, estrogen receptor; HR, hazard ratio.

By univariate Kaplan–Meier analyses, there were no significant associations between genetic defects (copy number, DNA sequence, or methylation) and survival, either in all patients (*P*=0.67) or among ER-positive patients (*P*=0.23). However, there was a marginal association between active/inactive expression subtype and survival among ER-positive patients (*P*=0.08) (Figure 3). Among the 76 patients with ER-positive disease, the active subtype was significantly associated with node-negative status (*χ* ^2^-test, *P*=0.02), but not with tumor subtype (*χ* ^2^-test, *P*=0.59), stage (*χ* ^2^-test, *P*=0.93), tumor size (*χ* ^2^-test, *P*=0.98), or presence of any genomic defect (Fisher’s exact test, *P*=1.0). Patients with active subtype tended to be older (average age 59.0 years) than inactive patients (average age 54.7 years), but the difference in age was not significant (two-sided pooled *t*-test, *P*=0.21). Because both previous literature and these univariate findings suggested that microenvironment subtype may predict survival among ER-positive (and/or hormone-treated) tumor patients, we conducted further Cox proportional hazards (multivariable) analyses adjusting for nodal status, intrinsic tumor subtype, tumor stage, tumor size, patient age in decade, and presence/absence of other cancer-adjacent genomic defects. In these multivariable analyses, active subtype was significantly associated with poorer survival among ER-positive cases, with a hazard ratio of 3.0 (confidence interval=1.8–5.1; *P*=0.04). The hazard ratio was not substantially altered (HR 2.9), but precision was reduced (confidence interval=0.9–9.7) when we excluded two stage IV cases.

## Discussion

In this study, the prevalence of genomic alterations in cancer-adjacent, benign-appearing breast tissue >2 cm from the tumor margin ranged from 10% for copy-number changes to >40% for RNA-detected mutations. The ~40% prevalence of detectable genomic defects exceeds the expected prevalence of breast cancer local/regional recurrences from clinical trial data 20 years following breast-conserving therapy,^9^ but is in range of estimates from other studies.^10,11^ However, in the current study, the presence of DNA copy number, methylation, or sequence defects was not associated with significant overall survival differences. These observations parallel recent reports showing that isolated tumor cells in axillary or sentinel nodes do not predict survival, whereas microscopic detection of a larger number of nodally infiltrated tumor cells is predictive of survival even with small tumor foci.^21^ It may be that cases with low cellular burden of unresected cancer cells do not increase recurrence rates and/or that ablation of these isolated tumor cells by radiation therapy is effective in preventing recurrences. Because the alterations observed in cancer-adjacent normal mirror the defects found in paired tumor tissue, although with a lower variant allele frequency, we speculate that the defects represent occult tumor cells. Previous studies have focused on the evidence of tumor cells in the lymph nodes, the current study extends these observations to include occult tumor cells and molecular abnormalities present in the local peritumor microenvironment.

Our results also show that the sensitivity of any given method to detect occult tumor cells, defined as malignant cells not observed/observable in pathological review, can vary between patients. DNA methylation was more sensitive in some patients and mutations were more sensitive in others. This apparent heterogeneity of genomic alterations among histologically benign-appearing specimens is consistent with the heterogeneity of defects in different subtypes of breast tumors.^19^ The effect of a methylation event in one patient may mimic that of a mutation event in another, and this molecular redundancy poses a technical challenge for biomarker development in both tumors and in cancer-adjacent tissue. With the advent of high-throughput molecular methods, this challenge is becoming surmountable, and here we used multiple genomic DNA and RNA platforms to assess whether ‘any genomic defect’ predicts progression. Although our data suggest that evidence of genomic defects by itself is unlikely to influence patient survival, normal tissue RNA expression profiles may have prognostic value.

RNA expression profiles of cancer-adjacent tissue predicted overall survival in this data set, consistent with the previous data showing prognostic value of RNA-based subtypes. The presence of two strong molecular subtypes in normal breast tissue was confirmed in this study, and it was also demonstrated that cancer-adjacent miRNA profiles mirror those of mRNA. This tendency for microRNA and mRNA subtypes to be correlated in normal breast contrasts with tumor, wherein microRNA and mRNA expression data appear to contribute distinct information or at least are non-overlapping.^19^ In cancer-adjacent tissue, differential expression of miR-200, a negative regulator of epithelial-to-mesenchymal transition, corresponds with the inactive mRNA subtype that also shows low expression of an epithelial-to-mesenchymal transition signature.^13^ Moreover, recent findings suggest that miR-200 members may inhibit metastasis and angiogenesis,^22^ consistent with observations that the inactive subtype is associated with better survival.

Despite a small sample size with limited follow-up, the survival analyses conducted here confirmed that expression subtypes of the cancer-adjacent normal tissue were associated with survival among ER-positive cases. We were unable to assess relapse as a separate outcome because of power constraints and population heterogeneity (stage I–IV, T1–T4). Because TCGA analyses do not allow for subsequent molecular studies on the same specimens, we were also unable to further investigate specific biological mechanisms. Future work should extend the rich genomic findings here to include more detailed characterization of microenvironment characteristics by immunohistochemistry (IHC) or other complementary technologies. Careful histopathological review and re-review are important in studies of normal tissue, particularly if normal tissue is a source of reference genomic DNA. Further studies following cohorts of patients with distinct normal breast tissue subtypes are needed, particularly studies with biospecimens available for mechanistic work, complete pathological re-review (herein we had three pathologists re-review tissue initially deemed cancer free, but emphasized 50 high priority cases with most comprehensive genomic data), and detailed relapse and overall survival data. Nonetheless, a growing body of evidence suggests that extratumoral microenvironment may have a role in progression of hormone receptor-positive disease.^13,18^

In summary, rich multi-platform data on histologically normal, breast cancer-adjacent tissue provide evidence that genomically altered cells are often present two or more centimeters from the tumor edge. Unless altered cells are also detected microscopically, the occult presence of such cells is unlikely to account for local recurrence following breast-conserving therapy, considering the high prevalence of these defects and consensus evidence that wider surgical margins provide no clinical benefit to patients.^12^ However, microenvironment subtypes in cancer-adjacent tissue may have prognostic value, and further investigation may elucidate mechanisms by which peritumoral stroma may contribute to survival.

## Materials and methods

### Cases and pathological assessment

Patients for this study provided informed consent to TCGA. Protocols were reviewed by Institutional Review Boards at all participating institutions. A total of 142 cases, excised 2 cm or more away from the tumor margin (precise distance and breast quadrant were not annotated), were analyzed (Supplementary Table 1). All samples were reviewed by a pathologist at TCGA’s Biospecimen Core Resource, of which 50 cases were selected for additional detailed pathological assessment. Frozen histological sections adjacent to sections used for molecular analyses were stained with hemotoxylin and eosin. Hemotoxylin and eosins were scanned and digital images were visually reviewed (K.H.A. and N.B.J.) and analyzed computationally (A.H.B). Slides were visually scored for the presence of tumor cells, presence of benign lesions, and percent area composed of epithelium/stroma/adipose, and a previously validated computational algorithm^15^ was applied to estimate percent composition. Some samples had limited or no visible epithelial content, and such slides were annotated ‘inconclusive’ (*n*=3, 6%) for evidence of tumor, but were retained in genomic analyses. Given high correlations between visual and digital assessment, digital estimates of percent area (epithelium/stroma/fat) were used for further analysis. Only one sample had evidence of tumor cellularity, and three samples (Figure 1f) were not interpretable due to poor slide quality. All clinical, histological slides, and molecular data are available through TCGA data portal (https://tcga-data.nci.nih.gov/tcga/) and bam files are available at CGHub (https://cghub.ucsc.edu/).

For whole-exome sequencing, 40 cases had tumor, blood, and adjacent normal samples for analysis. There were 40 cases with tumor, blood, and adjacent normal samples for analysis by the single-nucleotide polymorphism platform and an additional 71 with tumor and adjacent normal tissue but not blood normal. The other platforms analyzed only tumor and adjacent normal samples and include DNA methylation (*n*=118), mRNAseq for mutation analysis (*n*=103), mRNAseq for gene expression (*n*=107), mRNA by microarrays (*n*=60), and miRNAseq (*n*=102). See Supplementary Tables 1 and 2 for details. Survival analyses were conducted on 76 ER-positive cases (46=active, 30 inactive) with mRNA expression and survival data.

### Exome capture, sequencing, and alignment

All genomic data presented herein are available from TCGA Bioportal. Forty tumor samples, and matched blood and cancer-adjacent normal samples underwent exome capture and sequencing as previously described.^19^ In brief, exome libraries were generated using customized Agilent SureSelect All Exome v2.0 kit (Agilent Technologies, Santa Clara, CA, USA) or Nimblegen SeqCap EZ Human Exome v2.0 (Roche, Pleasanton, CA, USA) on the Illumina HiSeq2000 platform (Illumina, San Diego, CA, USA), and were sequenced to at least 10 Gbp. Samples achieving >70% coverage of the ~34 Mbp consensus coding sequence at 20× and a genotype concordance of >90% compared with high-density SNP array data were used for mutation detection and analysis. Somatic SNVs were identified using VarScan v2.2.6^23^ and SomaticSniper v0.7.3,^24^ and were filtered to remove read-mapping artifacts.^23^ Filter-passed somatic mutations were annotated using NCBI/ENSEMBL; only tier 1 mutations in coding regions, splice sites, or noncoding RNA genes were reported or validated by custom capture and deep resequencing (>150× average depth). The validation status for somatic mutations was defined by VarScan2, with the following parameters: minimum coverage=20; minimum variance frequency=0.10; somatic-*P*-value=0.05. Somatic mutations were measured in all three samples with ⩾20× coverage (Variant allele frequency, or VAF>10%), statistically significant (Fisher’s exact test; *P*-value<0.05) in the tumor, and absent (VAF<5%) in blood. Due to alignment bias and increased false positive rate for somatic indels, only somatic SNVs were used. Exome- and validation-sequencing experiments were combined for final calls, yielding average sequence depth of >300×.

### mRNA expression and microRNA sequencing and expression

For mRNA, Agilent custom 244 K whole-genome microarrays were hybridized and RNA library construction, sequencing, and analysis of sequence data were performed as described previously.^25^ RNA reads were aligned to the hg19 genome assembly using Mapsplice^26^ and gene expression was quantified for the transcript models corresponding to the TCGA GAF2.1, using RSEM^27^ and normalized within sample to the upper quartile. For further details on this data processing, refer to the Description file at the TCGA data portal under the V2_MapSpliceRSEM workflow. SNVs detected from DNA sequencing were interrogated in cancer-adjacent tissue RNA-sequencing using the program *UNCeqR.* ^28^ SNVs with at least two reads supporting the variant allele from DNA sequencing were defined as present and samples with at least two variant alleles present were scored as positive. Active/inactive mRNA subtypes were classified as described previously.^13^

For microRNAs, mature strand-sequencing data were generated as described.^19,29^ Two normalized reads per million (RPM) data matrices for 5p and 3p mature strands were input into non-negative matrix factorization unsupervised consensus clustering (v0.20.5, R v3.1.3):^30^ (a) 102 matched pairs of tumors and cancer-adjacent normal, then (b) only the 102 cancer-adjacent normal samples. For each RPM matrix, mature strands were ranked by RPM variance, and the most variant 25% (303) strands were clustered using the default Brunet algorithm, with 30 iterations for each step of the rank survey across the range 2–15 clusters, and 500 iterations, respectively, for each subsequent full clustering run. For the 102 normals, we assessed two-, four-, and eight-cluster solutions via consensus membership heatmaps, silhouette width profiles that we calculated from the consensus membership matrices as a measure of a sample’s typical/atypical membership status within a cluster, and concordance with mRNA active/inactive samples; we chose the two-cluster solution for consistency with prior knowledge about mRNA subtypes.^13^ Mature strands that were differentially abundant between these clusters were identified with a two-class unpaired SAM (samr v2.0)^31^ analysis in R v3.1.3, using as input the RPM abundance matrix for the 511 mature strands with a mean RPM of at least 1 in at least 10 of the 102 libraries, and with settings nperms=1,000, center.arrays=FALSE, testStatistic=‘wilcoxon’, and fdr.output=0.05. After filtering differentially abundant mature strands by requiring an absolute value fold change of at least 1.5 and a mean RPM of at least 25 in at least one of the two clusters, we generated barplots showing the largest positive and negative 25 fold changes. For the 90 mature strands passing the filtering noted above, we generated A normalized abundance (row-scaled log10(RPM+1) heatmap using pheatmap v1.0.2 (R Foundation for Statistical Computing, Vienna, Austria).

### DNA methylation profiling

DNA methylation profiling was carried out as described previously.^19^ In brief, we performed bisulfite conversion on 1 μg of DNA per sample and bisulfite-converted DNA was whole-genome amplified and enzymatically fragmented before hybridization to BeadChip arrays (HumanMethylation27 (HM27) and HumanMethylation450 (HM450), Illumina, San Diego, CA, USA). For HM27, mean fluorescence intensities of methylated (M) and unmethylated (U) bead types for each CpG locus were measured using Illumina BeadArray and extracted with Illumina GenomeStudio. For HM450, the level of DNA methylation at each CpG locus (β) was calculated as M/(M+U), ranging from 0 to 1. Of 50 samples with pathology data, 43 samples (HM450, *n*=22; HM27, *n*=21) were analyzed. We normalized data for batch effects using ComBat.^32^ Extent of occult tumor, defined as malignant cells not identified in pathological review, was assessed by regressing cancer-adjacent methylation on corresponding tumor methylation across 500 probes hyper-methylated in breast cancer. We interpreted a linear relationship between the two variables as suggestive of occult tumor cells. We identified samples with altered methylation based on high slope (>0.4). Slopes with low residual s.e. (<1) were considered to have occult tumor cells. Samples were ranked by slope and s.e. to order samples from high to low occult tumor cell probability and to generate a heatmap. A total of 1,000 probes with highest tumor–normal differences (500 hyper-methylated and 500 hypo-methylated) were clustered (separately for HM450 and HM27) using Heatplus in R/Bioconductor.

### Copy number

Segmented copy-number data for tumor, blood, and cancer-adjacent normal samples were collected as previously described.^19^ Forty samples had tumor, blood, and adjacent normal samples, and another 71 had tumor and adjacent normal tissue. Copy-number segments from cancer-adjacent normal were compared with those of tumor and blood. To be scored positive, a cancer-adjacent sample was required to have a segment with at least 50% reciprocal overlap (meaning the length of overlap between segments A and B is at least 50% of the length of A and 50% of the length of B) with a corresponding tumor, but no overlap with corresponding blood. Only segments with an absolute relative log2 copy-number change >0.1 were considered. All samples that scored positive were manually reviewed.

### Survival analysis

Associations between cancer-adjacent DNA defects or gene expression (mRNA/miRNA) subtype and overall patient survival were estimated using Kaplan–Meier curves and multivariable Cox proportional hazards models. Annotation of a DNA defect was defined as moderate or high for exome-sequencing mutation data, occult tumor contamination for methylation data, and ‘yes’ or ‘likely’ for CNV data (Supplementary Table 1). Multivariable survival models for DNA defects were fit among all tumors and for the subset of ER-positive tumors. Active/inactive expression subtype was evaluated in association with survival among ER-positive tumors and was adjusted for age (in decades: <40, 40 to <50, 50 to <60, 60 to <70, 70+), stage (I, II, or III/IV), size (T1, T2, T3, and T4), node status (positive versus negative), and tumor subtype (luminal A, luminal B, HER2, and basal like). Sensitivity analyses were conducted to evaluate whether adjustment for presence/absence of genetic defects (copy number, sequence or methylation) altered the association between extratumoral subtype and survival and to evaluate whether exclusion of stage IV cases altered estimates of the hazard ratio. Survival curves were censored at 10 years because few patients had clinical follow-up data beyond this time.

## Figures and Tables

**Table 1:** Evidence of genomic abnormalities in normal breast tissue by data type in The Cancer Genome Atlas Project

*Data type*	N *(%)*
*microRNA* a	
Normal like	97 (95)
Tumor like	5 (5)
	
*mRNA expression* a *(microarray)*	
Normal like	56 (93)
Tumor like	4 (7)
	
*mRNA expression* a *(RNA-Seq)*	
Normal like	95 (89)
Tumor like	12 (11)
	
*mRNA sequence (RNA-Seq)*	
No mutations detected	58 (56)
2+ mutations detected	45 (44)
	
*Exome sequencing* b	
High	4 (10)
Moderate	6 (15)
Low	3 (8)
None	27 (68)
	
*Copy number triplets* c	
Evidence of tumor	4 (10)
Small evidence of tumor	8 (20)
Normal	28 (70)
	
*Copy number pairs* d	
Likely evidence of tumor	6 (8)
Normal	65 (92)
	
*DNA methylation* e	
Occult tumor	18 (15)
Field effect	43 (36)
Normal	57 (48)

Abbreviations: mRNA, messenger RNA; RNA-Seq, RNA sequencing; VAF, variant allele fraction.

a For expression-based RNA and microRNA calls, abnormal is, respectively, defined by having a tumor-like PAM50 class and by clustering with tumor samples.

b For mutations from exome sequencing, high is defined as at least two mutations and at least 50% with VAF>1% in the adjacent normal, moderate as at least 2 mutations but no >50% with VAF>1%, low as at least 2 mutations but no >10% with VAF>1%, and none as <2 mutations with VAF>1%.

c For copy number triplets, evidence of tumor is defined as >100,000 bp of copy-number alterations shared between tumor and adjacent normal, small evidence is defined as 1,000–99,999 bp of copy-number alterations shared between tumor and adjacent normal.

d For copy-number pairs, we only had tumor and adjacent normal, likely evidence of tumor is defined as >100,000 bp of copy-number alterations shared between tumor and adjacent normal; however, blood normal is needed for confirmation, which was not available.

e DNA methylation patterns were classified as reflecting occult tumor, field cancerization, or normal as described in the Materials and Methods section.
