# Supplementary figures and images for: DNA defects, epigenetics, and gene expression in cancer-adjacent breast: a study from The Cancer Genome Atlas
Source: NPJ Breast Cancer. 2016 May 4;2:16007–. doi: 10.1038/npjbcancer.2016.7 (PMC5515343; doi:10.1038/npjbcancer.2016.7)

A

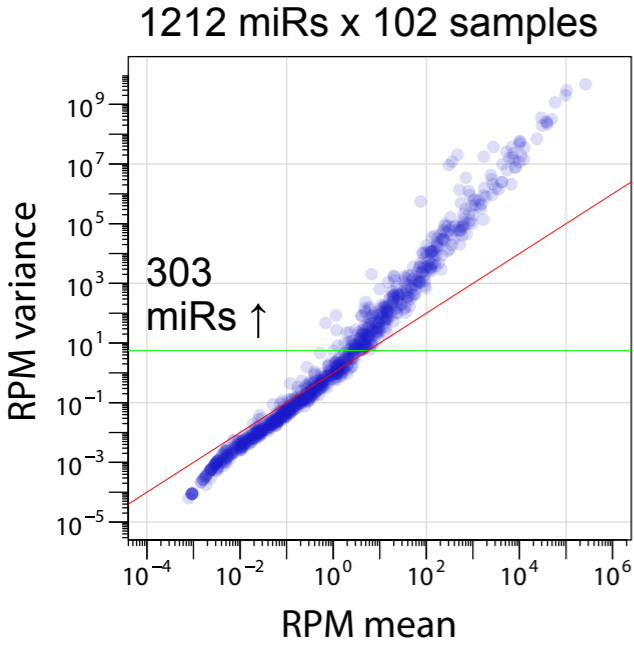

B

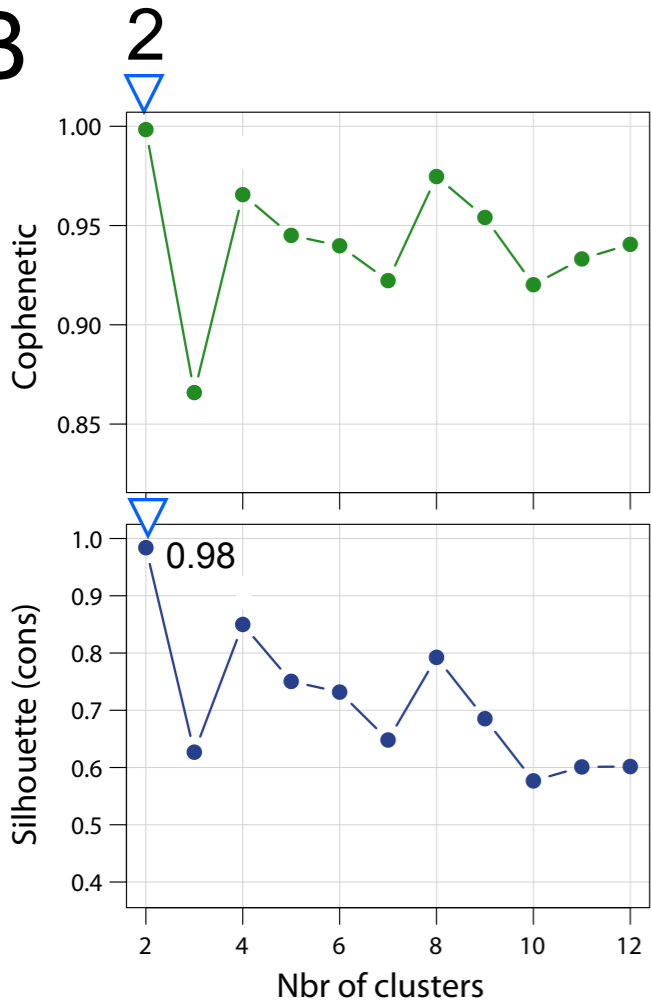

C

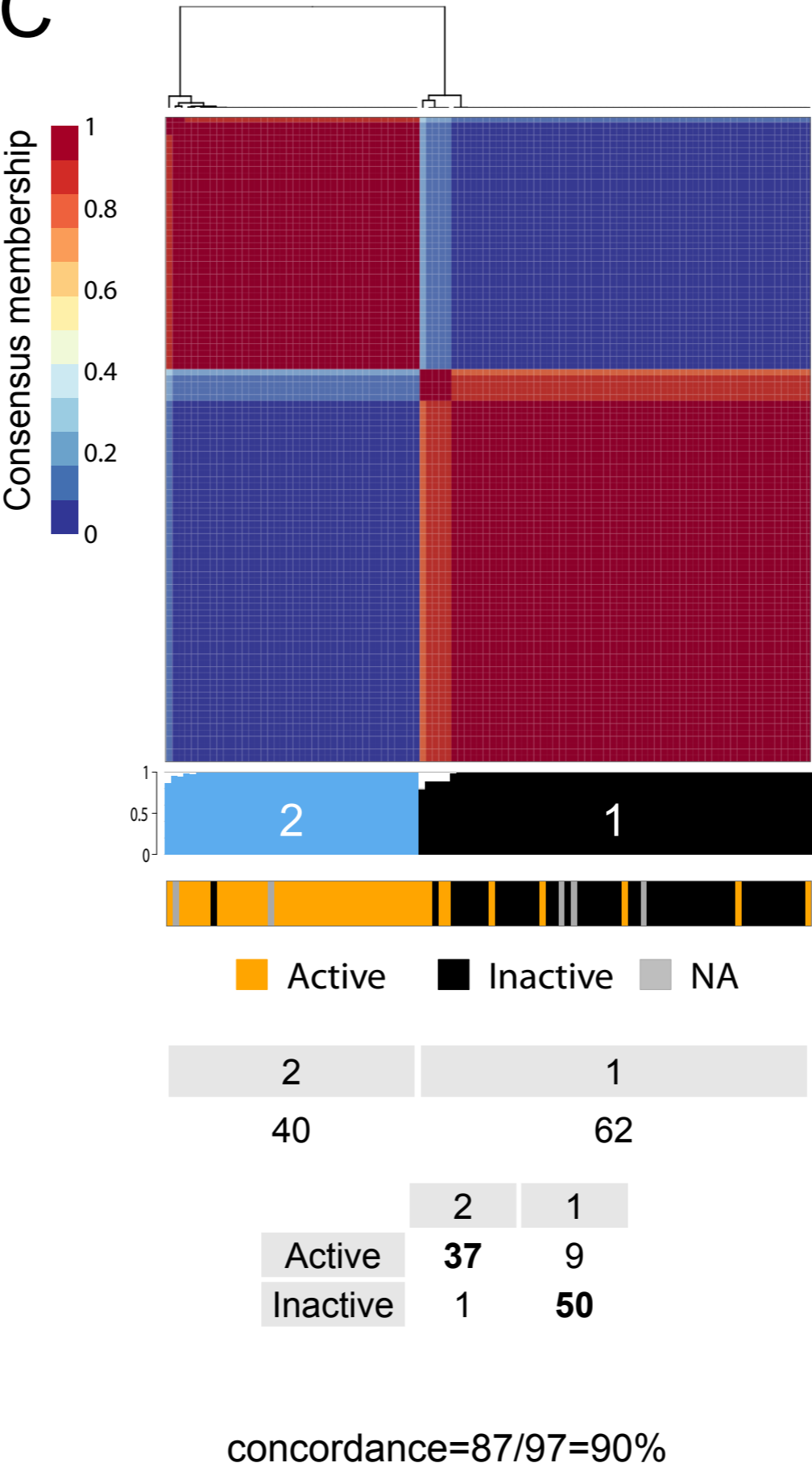

Supplement: Supplementary Figure 1 [file npjbcancer20167-s1.pdf]
